# Characterizing a standardized BioPart for PVQ-specific expression in *C. elegans*

**DOI:** 10.17912/micropub.biology.000870

**Published:** 2023-06-22

**Authors:** Sarah AlHarbi, Christian Frøkjær-Jensen

**Affiliations:** 1 King Abdullah University of Science and Technology (KAUST), Biological and Environmental Science and Engineering Division (BESE), KAUST Environmental Epigenetics Program (KEEP), Thuwal, 23955-6900, Saudi Arabia

## Abstract

Synthetic biology relies on standardized biological parts (BioParts), and we aim to identify cell-specific promoters for every class of neuron in *C. elegans* . Here, we characterize a short BioPart (P *nlp-17* , 300 bp) for PVQ-specific expression. P *nlp-17 ::mScarlet* showed bright, persistent, and specific expression in hermaphrodite and male PVQ neurons from multicopy arrays and single-copy insertions starting from the comma stage. We generated standardized P *nlp-17* cloning vectors with *gfp* and *mScarlet* compatible with single-copy or array expression for PVQ-specific transgene expression or identification. To facilitate gene synthesis, we have incorporated P *nlp-17* as a standard BioPart in our online transgene design tool (www.wormbuilder.org/transgenebuilder).

**Figure 1. Expression and Characterization of P nlp-17 in PVQ Neurons of C. elegans:**
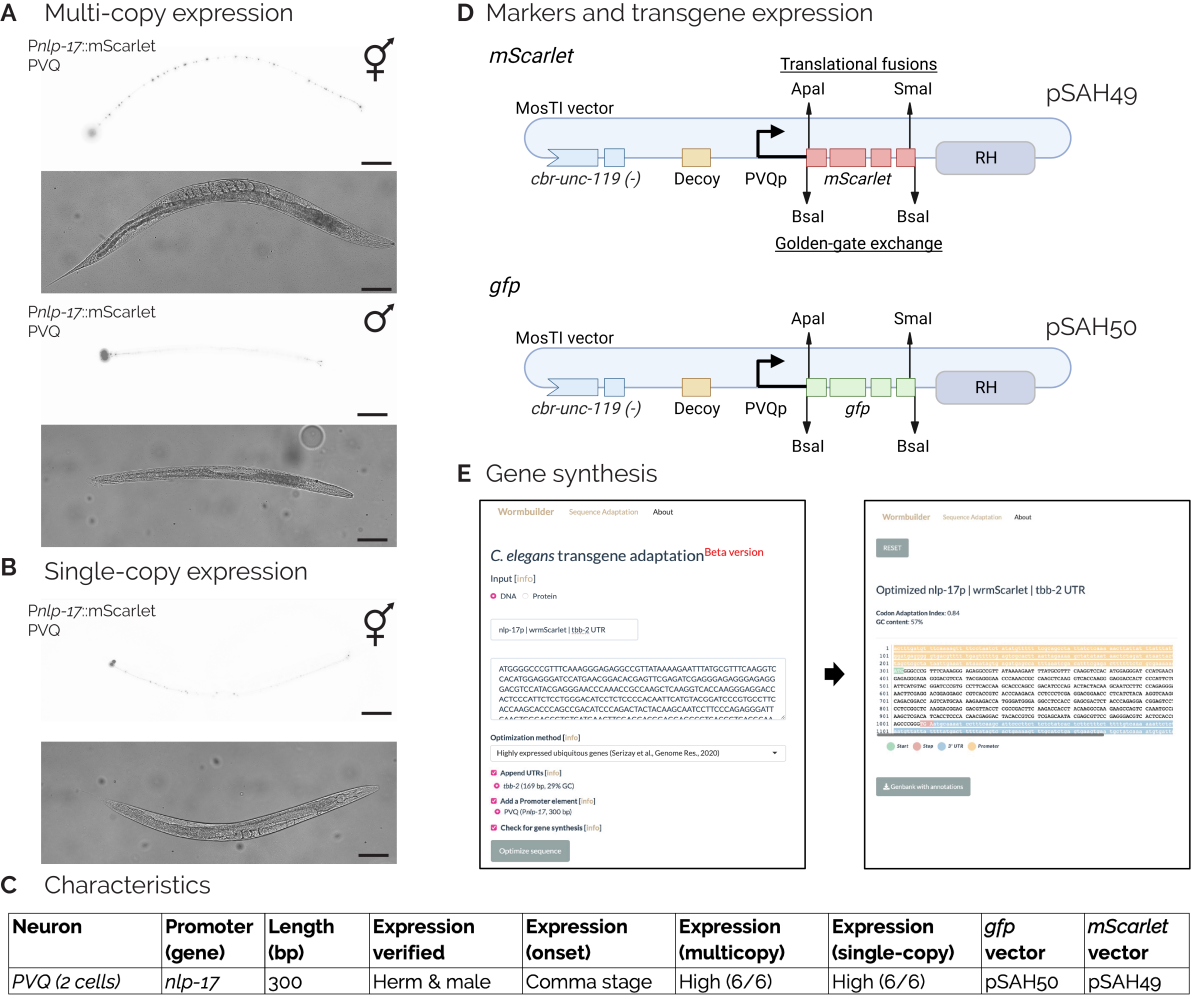
**A.** P *nlp-17 ::mScarlet:: tbb-2* 3'UTR expression is specific to PVQ neurons from extrachromosomal arrays in hermaphrodites (top panel) and males (bottom panel). **B.** Single-copy P *nlp-17 ::mScarlet* transgenes inserted by MosTI (El Mouridi et al., 2022) are easily visible in hermaphrodites. 20x magnification. Scale bars: 100 µm. **C.** Characteristics and quantification of the short 300 bp *nlp-17* promoter. **D.** Schematic of standard BioPart expression vectors for PVQ expression. Unmodified *gfp* or *mScarlet* vectors can be used to identify PVQ neurons. Standard restriction sites allow cell-specific expression with N-terminal (ApaI) and C-terminal (SmaI) fluorophore fusions or complete fluorophore exchange (BsaI). The vectors are compatible with single-copy insertions (MosTI) and expression from extra-chromosomal arrays. **E.** The short 300 bp promoter is easily synthesized and has been included as a standard feature in an online tool for optimizing transgene design and synthesis (www.wormbuilder.org/transgenebuilder) (Vargas-Velazquez, El Mouridi, Alkhaldi, and Frøkjær-Jensen C, manuscript in preparation).

## Description

Synthetic biology is an interdisciplinary field that aims to apply engineering principles to the design and construction of biological systems. One key aspect of synthetic biology is the use of standardized and interchangeable biological parts, known as BioParts, to build biological circuits (Knight, 2003; Shetty et al., 2008) . Standardization enables compatibility between different BioParts, improving reproducibility and enabling easy design with the assembly of multiple BioParts in a single reaction (Engler et al., 2008; Gibson et al., 2009; Kok et al., 2014; Li and Elledge, 2012) . Ideally, the properties of BioParts should be well-characterized and behave reproducibly in different contexts, similar to how electrical parts ( *e.g.* , resistors and capacitors) behave in predictable ways in electrical circuits. While BioParts have been extensively used in single-celled organisms, they are less commonly used in multicellular organisms (Malcı et al., 2022). *C. elegans* is an exception, with standardized vector kits and detailed documentation distributed informally to the scientific community by the Fire laboratory (https://www.addgene.org/kits/firelab/) playing an essential role in facilitating transgene design and reproducibility between different laboratories.

A collection of BioParts composed of neuron-specific promoters would be expected to be useful for many different experiments, e.g., as a starting point for genetic screens (e.g., Johnston and Hobert, 2003; Shen et al., 2004), imaging neuronal activity (e.g., Chalasani et al., 2007; Suzuki et al., 2003), or controlling neuronal activity using optogenetics (Nagel et al., 2005) . The *C. elegans* genome is compact with relatively short intergenic regions (Narasimhan et al., 2015) , and short neuronal promoters (<300 bp) are frequently sufficient to drive cell-specific expression in transcriptional reporter assays (e.g., Etchberger et al., 2009; Wenick and Hobert, 2004; Froehlich and Rajewsky, 2023). Our initial experiments are based on a curated list of neuron-specific promoters (Lorenzo et al., 2020) , and we aim to identify additional neuron-specific promoters from a comprehensive neuronal atlas based on single-cell RNA-seq (Taylor et al., 2021) . We have set out to identify and characterize neuron-specific promoters that are (1) short to facilitate gene synthesis and (2) relatively strong to allow controlled expression from single-copy transgenes. We expect that well-characterized BioParts will facilitate the design and synthesis of genes for use in biological circuits. Here, we describe a standardized BioPart for cell-specific expression in *C. elegans* PVQ neurons, a class of interneurons involved in regulating locomotion and feeding behaviors (Frank et al., 2003) .

In this study, we focused on a shortened version of the *nlp-17* promoter, which is specific to PVQ neurons (Lorenzo et al., 2020) . To determine expression, we synthesized a fluorescent reporter construct containing 300 bp of the *nlp-17* promoter fused to a red fluorophore ( *mScarlet* ) and a standard 3' UTR ( *tbb-2* ) (P *nlp-17* :: *mScarlet* ). Transgenic *C. elegans* with P *nlp-17* :: *mScarlet* in extra-chromosomal arrays (Mello et al., 1991) displayed consistently bright red fluorescence (intensity = 6, on a scale from 1 (dim) to 6 (bright), see methods for details), in two bilaterally symmetric neurons consistent with PVQ neuron position and morphology ( **Figure 1A** ). We included a piRNA interference (piRNAi) fragment targeting *him-5* in the injection mix (Priyadarshini et al., 2022) , which allowed us to verify PVQ-specific expression in males ( **Figure 1A** ). To generate standardized reagents, we cloned the *nlp-17* promoter into two vectors, containing either *gfp* or *mScarlet* , that are compatible with single-copy transgene insertions using MosTI (El Mouridi et al., 2022) . With these reagents, we generated a single-copy *mScarlet* reporter insertion and observed readily visible expression on a fluorescence dissection microscope at high magnification (intensity = 6 out of 6) (Figure **1B** ). We characterized the onset of fluorescence in transgenic embryos and observed expression beginning in the comma stage. The main characteristics of the *nlp-17* promoter are summarized in **Figure 1C** .

The two standard vectors, *mScarlet* (pSAH49) and *gfp* (pSAH50) should be useful for identifying PVQ neurons or for cell-specific transgene expression, and we have deposited the plasmids with Addgene ( **Figure 1D** ). The constructs were engineered with restriction sites for N-terminal (ApaI) and C-terminal (SmaI) transgene fusions or exchange of the fluorophore (BsaI). The vectors contain a 5' decoy sequence to minimize misexpression (A. Fire, personal communication), are compatible with MosTI single-copy insertion, and can be used for extra-chromosomal arrays. The short promoter is particularly useful for gene synthesis; to facilitate transgene design, we have incorporated the *nlp-17* promoter sequence into our online transgene design app (www.wormbuilder.org/transgenebuilder) (Vargas-Velazquez, El Mouridi, Alkhaldi, and Frøkjær-Jensen, manuscript in preparation) ( **Figure 1 E** ).

These design principles and reagents constitute the first of a series of planned BioParts for neuronal transgene expression. Ultimately, we aim to generate compatible and easily synthesized BioParts that allow cell-specific expression in every neuronal class, with the goal of easy cell identification and genetic control of every cell with optogenetics tools (Zhang et al., 2007) .

## Methods

**Molecular biology**

We generated non-clonal synthetic transgenes by gene synthesis (Twist Bioscience, CA, USA) and clonal plasmids by Golden-Gate cloning (Engler et al., 2008) using Esp3I (New England Biolabs). Clonal plasmids were validated by restriction digest. We shortened the *nlp-17* promoter tested by Lorenzo et al. (2020) from 339 basepairs to 300 basepairs and removed any BsaI, Esp3I, ApaI, SmaI, and EcoRV restriction sites. We also modified the last four basepairs of the promoter to the consensus start site (aaaaATG).

**Quantifying promoter strength**

We quantified the fluorescence intensity of mScarlet reporter constructs in transgenic animals with stable extra-chromosomal arrays (typically in the F2 or F3 generation) or single-copy MosTI insertions at the *ttTi5605* location on Chr. II. We quantified the fluorescence on a dissection stereo microscope (ZEISS, Olympus SZX2-FOF), objectives 1x and 10x, using an mTomato filter set, and an LED light source (X-Cite XYLIS, XT720L), or on an upright compound microscope (LEICA DM2500 LED), 40x oil immersion objective, with a Rhodamine filter set (Leica 11504205), and a mercury metal halide bulb (Leica EL6000). We quantified fluorescence intensity visually in ten L4 animals at different magnifications. We scored both males and hermaphrodites with no obvious differences in expression. The quantification was performed by eye and scored on a scale from 1 to 6, with 1 being the dimmest and 6 being the brightest. The following criteria were used for scoring. **Dissection microscope** . Score = 6: fluorescence visible with 1x objective and zoom = 1 (lowest). Score = 5: fluorescence visible with 1x objective and zoom = 8 (highest). Score = 4: fluorescence visible with 10x objective and zoom = 1. Score = 3: fluorescence visible with 10x objective and zoom = 8. **Compound microscope** . Score = 2: fluorescence visible with 20x air objective. Score = 1: fluorescence visible with 40x oil immersion objective. Score = 0: fluorescence not visible at 40x oil immersion objective.

**Onset of promoter expression**

We screened at least 15 embryos from transgenic animals at various stages (gastrula, comma, 1.5-fold, 2-fold, and 3-fold) on NGM plates for the first visible expression on a dissection microscope. We observed no obvious difference in the onset of expression between transgenic animals with extra-chromosomal arrays and single-copy transgenes.

**Microscopy**

Transgenic animals were mounted on 2% agarose pads and anesthetized with an M9 solution containing 50 mM of sodium-azide. We imaged animals with a Leica THUNDER Imaging System, with a 20x oil immersion objective, capturing four image stacks, two for hermaphrodites and two for males. After imaging, we generated maximum intensity projection on Leica LAS X software. We carefully screened for misexpression in additional neurons other than PVQ but did not observe any.

**Transgenic animals**

Extra-chromosomal arrays. Transgenic animals carrying extra-chromosomal arrays were generated by injecting into *unc-119* animals ( CFJ42 ). The injection mix contained 10 ng/µl of a non-clonal PVQ specific fragment *nlp-17 p::mScarlet:: tbb-2 3'UTR* (Twist Bioscience), 10 ng/µl pCFJ108 ( *ce- unc-119* rescue) linearized with ApaLI, 10 ng/µl pCFJ782 (hygromycin resistance) linearized with EcoRV (Radman et al., 2013) , 10 ng/µl pMNK54 (piRNAi targeting *him-5* ) linearized with ApaLI, and 60 ng/µl GeneRuler 1 kb plus DNA ladder (ThermoFisher SM1331) for a final concentration of 100 ng/µl. After injection, three independent stable transgenic lines were maintained and scored for mScarlet expression.

MosTI insertions. Single-copy transgenes with pSAH49 ( *nlp-17 p* :: *mScarlet* :: *tbb-2* 3'UTR) were inserted by MosTI into Chr II ( CFJ42 , *ttTi5605* site) following standard protocols (El Mouridi et al., 2022) . The injection mix contained 10 ng/µl pSAH49 ( *nlp-17 p* :: *mScarlet* ) MosTI targeting vector, 10 ng/µl pSEM238 (histamine negative selection) (El Mouridi et al., 2021) , 15 ng/µl pSEM318 (MosTI sgRNA), 10 ng/µl pSEM231 ( *mlc-p1* :: *gfp* , co-injection marker) (Mouridi et al., 2020), 15 ng/µl pCFJ782 (hygromycin resistance) (Radman et al., 2013) , 15 ng/µl pMDJ231 (heat-shock Cas9) linearized with ApaLI, 25 ng/µl GeneRuler 1 kb plus DNA ladder (ThermoFisher SM1331) for a final concentration of 100 ng/µl. After injection, the animals were grown at 25°C, and hygromycin was added to the plates on day 3. When the bacterial lawn was nearly exhausted, we heat-shocked the plates with transgenic animals in a 30°C air incubator for 20 hours. The day after the heat shock, the plates were transferred to new NGM plates by chunking. Transgenic animals with a single-copy insertion were identified four days after the heat shock based on *unc-119* rescue. A single clonal rescued worm with no visible fluorescence from the co-injection marker was picked to generate a stable line.

**Software**

The online application for designing transgenes is available at www.wormbuilder.org/transgenebuilder. A manuscript detailing the application is in preparation by the authors (Vargas-Velazquez, El Mouridi, Alkhaldi, and Frøkjær-Jensen). The version described in this manuscript has been archived at https://github.com/christianfj/transgenebuilder_v2023_06_13 and available as Extended Data.

*In silico* molecular biology design was performed with "A plasmid Editor" (ApE) (Davis and Jorgensen, 2022) . Schematics for the expression vectors were created with BioRender (Biorender.com) . The figure was generated with Adobe Illustrator (version 27.4).

## Reagents

**Plasmids**

pSAH49 - *nlp-17 p::mScarlet::tbb-* 2 (MosTI vector) (Addgene #200325)

pSAH50 - *nlp-17 p::gfp:: tbb-2* (MosTI vector) (Addgene #200326)

pMNK54 - *him-5* piRNAi fragment (Addgene #159818) (Priyadarshini et al., 2022)

pCFJ108 - *cbr- unc-119* (+) rescue marker (Addgene #200367)

pSEM238 - *snt-* 1p *::ce-HisCl1:: rpl-3* (Addgene #161515) (El Mouridi et al., 2021)

pSEM318 - *rpr-1p::sgRNA* targeting the *ttTi5605* location Chr. II (Addgene #159822)

pSEM231 - *mlc-1p::gfp::cbr- tbb-2* (Addgene #159897) (Mouridi et al., 2020)

pCFJ782 - *rps-0p::hygromycinR:: rps-27* (Addgene #190933) (Radman et al., 2013)

pMDJ231 - *hsp-16.41p::cas9:: gpd-2 ::tagRFP-t:: smu-1* (Addgene #191382)

**Strains**

N2 - (wildtype)

CFJ42 - *kstSi42* [ *unc-119* (p1, spc2)(-)] II; *unc-119* ( *ed3* )] III (available from CGC)

**Promoter sequence**

**Table:** 

WormBase ID	Gene name	Promoter sequence (modifications in upper case, consensus start site in bold)
WBGene00003755	*nlp-17*	actttgatgtttcaaaagttttcctaatctatatgttttttcgcagcctatt atctcaaaaacttattatttatttatttatctaggttattacgggaatggat gagggggtgacgtttttgagtttttgagtcgcacttaattagaaaagctata taataactctagatataattactcgActcacttaatagccttgatagctcgc tataattgaaatataaatagtgagatgagccatttaaatcgacatttcgaga cttttttctcgcgaaaaagtcaggctttttcacaga **aaaaATG**

**Transgene sequences**

**Table:** 

Plasmid	Transgene	DNA sequence (coding sequence in upper case)
pSAH49	* decoy:: nlp-17 p:: * *mScarlet::tbb-* 2	gatatcaaaaatgattacgccaagctgtaagtttaaacatgatctactaactaactattctcatttaaattttcagagcttaaaaatggctgaaatcactcacaacgatggatacgctaatgccactttgatgtttcaaaagttttcctaatctatatgttttttcgcagcctattatctcaaaaacttattatttatttatttatctaggttattacgggaatggatgagggggtgacgtttttgagtttttgagtcgcacttaattagaaaagctatataataactctagatataattactcgactcacttaatagccttgatagctcgctataattgaaatataaatagtgagatgagccatttaaatcgacatttcgagacttttttctcgcgaaaaagtcaggctttttcacagaaaaaagagaccaaaaATGGGGCCCGTTTCAAAGGGAGAGGCCGTTATAAAAGAATTTATGCGTTTCAAGGTCCACATGGAGGGATCCATGAACGGACACGAGTTCGAGATCGAGGGAGAGGGAGAGGGACGTCCATACGAGGGAACCCAAACCGCCAAGgtgagttttattatcgattttcgggattttctacttgtaattaccttttgtttttaataagttgcttttttccagCTCAAGGTCACCAAGGGAGGACCACTCCCATTCTCCTGGGACATCCTCTCCCCACAATTCATGTACGGATCCCGTGCCTTCACCAAGCACCCAGCCGACATCCCAGACTACTACAAGCAATCCTTCCCAGAGGGATTCAAGTGGGAGCGTGTCATGAACTTCGAGGACGGAGGAGCCGTCACCGTCACCCAAGgtaggttttcagtttcaatttttgttgcaactacctcaactctatgttttcagACACCTCCCTCGAGGACGGAACCCTCATCTACAAGGTCAAGCTCCGTGGAACCAACTTCCCACCAGACGGACCAGTCATGCAAAAGAAGACCATGGGATGGGAGGCCTCCACCGAGCGACTCTACCCAGAGGACGGAGTCCTCAAGGGAGACATCAAGgtaaatttttcaagcttctaaattccaaaataaaccattttaatttcttaattccagATGGCCCTCCGGCTCAAGGACGGAGGACGTTACCTCGCCGACTTCAAGACCACCTACAAGGCCAAGAAGCCAGTCCAAATGCCAGGAGCCTACAACGTCGACCGTAAGCTCGACATCACCTCCCACAACGAGGACTACACCGTCGTCGAGCAATACGAGCGTTCCGAGGGACGTCACTCCACCGGAGGAATGGACGAGCTCTACAAGCCCGGGTGAtggtctctgagctatgcaaaatcctttcaagcattcccttcttctctatcactcttctttctttttgtcaaaaaattctctcgctaatttatttgcttttttaatgttattattttatgactttttatagtcactgaaaagtttgcatctgagtgaagtgaatgctatcaaaatgtgattctgatatc
pSAH50	* decoy:: nlp-17 p:: * *gfp::tbb-* 2	gatatcaaaaatgattacgccaagctgtaagtttaaacatgatcttactaactaactattctcatttaaattttcagagcttaaaaatggctgaaatcactcacaacgatggatacgctaatgccactttgatgtttcaaaagttttcctaatctatatgttttttcgcagcctattatctcaaaaacttattatttatttatttatctaggttattacgggaatggatgagggggtgacgtttttgagtttttgagtcgcacttaattagaaaagctatataataactctagatataattactcgactcacttaatagccttgatagctcgctataattgaaatataaatagtgagatgagccatttaaatcgacatttcgagacttttttctcgcgaaaaagtcaggctttttcacagaaaaaagagaccaaaaATGGGGCCCTCTAAGGGCGAAGAATTGTTCACGGGAGTAGTCCCAATCTTAGTCGAGCTTGACGGAGgtgagttttattatcgattttcgggattttctacttgtaattaccttttgtttttaataagttgcttttttccagATGTTAATGGCCACAAGTTTTCCGTGTCTGGAGAAGGAGAAGGCGACGCTACCTACGGTAAGCTGACCCTGAAGTTCATTTGCACTACTGGCAAGTTACCAGTACCATGGCCGACCCTGGTGACAACATTTTGCTACGGGGTTCAATGCTTCTCCAGATACCCAGgtaggttttcagtttcaatttttgttgcaactacctcaactctatgttttcagATCATATGAAAAGACACGACTTTTTTAAATCCGCCATGCCAGAGGGTTACGTTCAAGAACGTACCATTTTTTTTAAGGACGATGGTAACTACAAGACACGTGCGGAGGTTAAATTTGAAGGCGATACTCTCGTGAACCGTATTGAGCTCAAGgtaaatttttcaagcttctaaattccaaaataaaccattttaatttcttaattccagGGAATCGATTTCAAGGAAGATGGAAACATTTTGGGCCACAAGCTTGAATATAATTATAATAGTCATAATGTCTACATTATGGCCGACAAACAAAAAAACGGTATTAAGGTTAACTTCAAGATTCGCCACAATATCGAAGATGGATCTGTCCAACTTGCTGACCACTACCAACAAAATACTCCAATTGGAGATGGACCGGTTTTGCTTCCAGATAACCACTACCTTTCCACTCAGTCTGCCCTCTCTAAGGATCCGAATGAAAAACGTGACCATATGGTTCTTTTGGAGTTCGTTACCGCAGCAGGAATTACTCACGGAATGGATGAACTGTACAAGCCCGGGTGAtggtctctgagctatgcaaaatcctttcaagcattcccttcttctctatcactcttctttctttttgtcaaaaaattctctcgctaatttatttgcttttttaatgttattattttatgactttttatagtcactgaaaagtttgcatctgagtgaagtgaatgctatcaaaatgtgattctgatatc

## Extended Data

Description: Online application for designing transgenes. . Resource Type: Software. DOI: 10.22002/qs7eh-g0669
